# Rivaroxaban versus warfarin: differential effects on oxidative stress and fibrinolytic markers in atrial fibrillation

**DOI:** 10.3389/fphar.2026.1685101

**Published:** 2026-01-19

**Authors:** Helton Jose Reis, Luana Bernardes Xavier Costa, Gabriela Lopes Martins, Rita Carolina Figueiredo Duarte, Luma Clara Martins Costa, Estêvão Lanna Figueiredo, Francisco Rezende Silveira, Nathália Greco Coelho, Maria das Graças Carvalho, Luciene Bruno Vieira, Edna Afonso Reis, Karina Braga Gomes, Cláudia Natália Ferreira

**Affiliations:** 1 Departamento de Farmacologia, Instituto de Ciências Biológicas (ICB), Universidade Federal de Minas Gerais, Belo Horizonte, Minas Gerais, Brazil; 2 Faculdade de Farmácia, Universidade Federal de Minas Gerais, Belo Horizonte, Minas Gerais, Brazil; 3 Hospital Lifecenter, Belo Horizonte, Minas Gerais, Brazil; 4 Hospital Semper, Belo Horizonte, Minas Gerais, Brazil; 5 Departamento de Estatística, Instituto de Ciências Exatas (ICEx), Universidade Federal de Minas Gerais, Belo Horizonte, Minas Gerais, Brazil; 6 Colégio Técnico, Universidade Federal de Minas Gerais, Belo Horizonte, Minas Gerais, Brazil

**Keywords:** atrial fibrillation, fibrinolytic system, oxidative stress, rivaroxaban, warfarin

## Abstract

**Background:**

Atrial fibrillation (AF) is a cardiac arrhythmia characterized by disorganized atrial electrical activity, resulting in ineffective mechanical contraction and a heightened propensity for intra-atrial thrombus formation. The underlying pathophysiology is multifactorial, involving a complex interplay of pro-fibrotic, inflammatory, and pro-thrombotic pathways, notably oxidative stress and dysregulation of the fibrinolytic system. Given that these mechanisms remain incompletely elucidated, this study sought to investigate the association between biomarkers of oxidative stress and antifibrinolytic activity in AF patients treated with the oral anticoagulants warfarin or rivaroxaban, in comparison to a healthy control cohort.

**Methods:**

A total of 85 AF patients—38 on rivaroxaban and 47 on warfarin—were enrolled alongside 62 matched healthy controls. Cellular metabolic activity was assessed via MTT [3-(4,5-Dimethylthiazol-2γl)-2,5-Diphenyl Tetrazoline Bromide] assay measured by spectrophotometry. Serum concentrations of thiobarbituric acid reactive substances (TBARS, a marker of lipid peroxidation), plasminogen activator inhibitor-1 (PAI-1), and thrombin-activatable fibrinolysis inhibitor (TAFI) were quantified using enzyme-linked immunosorbent assay (ELISA).

**Results:**

The rivaroxaban group exhibited significantly greater MTT absorbance, indicative of enhanced cellular metabolic activity, and significantly lower circulating TAFI levels compared to the warfarin group.

**Conclusion:**

These results suggest that in patients with AF, rivaroxaban may provide pleiotropic benefits beyond anticoagulation, potentially by augmenting cellular antioxidant mechanisms and suppressing antifibrinolytic activity.

## 1 Introduction

Atrial fibrillation (AF) is the most common cardiac arrhythmia encountered in clinical practice. It is characterized by rapid and disorganized atrial excitation, resulting in irregular ventricular activation (Westerman and Wenger, 2019). The presence of AF is strongly associated with increased cardiovascular morbidity and mortality (Meyre et al., 2025). With a globally aging population, the projected prevalence of AF is expected to rise significantly over the coming decades, posing a substantial economic burden on healthcare systems (Ki et al., 2011).

AF is intrinsically linked to an elevated risk of thromboembolic events. Recent studies have demonstrated that the increasing use of oral anticoagulants has contributed to a concomitant reduction in AF-related stroke incidence (Cowan et al., 2018; Freedman, 2018; Wallentin et al., 2025). Accordingly, a key therapeutic strategy in the management of AF is the use of oral anticoagulation to prevent thromboembolic events.

Historically, the CHA_2_DS_2_-VASc score has been the principal tool for thromboembolic risk stratification and for guiding the initiation of anticoagulation therapy in patients with AF (Vitali et al., 2019). In a recent development, Teppo et al. proposed a modified scoring system, the CHA_2_DS_2_-VA score, which omits female sex as an independent risk factor in AF patients (Teppo et al., 2024). Due to limitations associated with traditional anticoagulants such as warfarin—including dietary interactions, variable dosing, and the requirement for regular monitoring—direct oral anticoagulants (DOACs) have been increasingly adopted in clinical practice owing to their more predictable pharmacokinetics and ease of use (Lee, 2016). Rivaroxaban, a representative DOAC, exerts its anticoagulant effect through direct inhibition of activated factor X (FXa). Emerging evidence also supports its potential pleiotropic effects, including anti-inflammatory activity, attenuation of vascular remodeling, regression of atherosclerotic plaques, and inhibition of tissue fibrosis (Spronk et al., 2014; Van Gorp and Schurgers, 2015; Terry et al., 2016; Katoh et al., 2017).

Oxidative stress plays a critical role in the pathophysiology of AF, being associated with atrial enlargement, arrhythmia prevalence, and increased risk of recurrence (Ghasemzadeh et al., 2014; Tahhan et al., 2017; Huang et al., 2019; Rafaqat et al., 2023). Tanhan et al. demonstrated that elevated oxidative stress levels were associated with a 30% increase in AF incidence and prevalence (Tahhan et al., 2017). Moreover, recent findings suggest that FXa inhibitors, such as rivaroxaban, may reduce oxidative stress by inhibiting the generation of reactive oxygen species (Woźniak et al., 2020; Pfen et al., 2024).

Fibrinolysis, the physiological process responsible for the degradation of fibrin clots, is essential in maintaining vascular patency (Lin et al., 2020). The interplay among blood stasis, endothelial injury, and alterations in fibrinolysis constitutes a well-established mechanism underlying thromboembolic events in AF (Freestone and Lip, 2008). While several studies have reported elevated levels of anti-fibrinolytic markers such as plasminogen activator inhibitor-1 (PAI-1) (Adams and Huntington, 2006) and thrombin-activatable fibrinolysis inhibitor (TAFI) (Ząbczyk et al., 2011) in AF patients, the precise role of impaired fibrinolysis in AF-associated thromboembolism remains insufficiently elucidated (Tóth et al., 2017).

The complex pathophysiology of AF is characterized by the interplay of multiple pathways, notably oxidative stress and inflammation, which drive atrial electrical and structural remodeling, fibrosis, and elevated thromboembolic risk (Gutierrez and Van Wagoner, 2015; Ellinghaus et al., 2016). The fibrinolytic system is a key modulator of these inflammatory processes. Accordingly, alterations in fibrinolytic biomarkers, including TAFI and PAI-1, have been associated with both oxidative stress and inflammation in the context of cardiovascular disease (Pang et al., 2017). In light of emerging evidence on the pleiotropic effects of rivaroxaban, we hypothesized that this agent would favorably modulate plasma levels of key oxidative stress and fibrinolytic markers in patients with AF.

Therefore, the present study was designed to evaluate the association between oxidative stress and antifibrinolytic biomarkers and the presence of AF in patients undergoing treatment with oral anticoagulants (warfarin or rivaroxaban), in comparison to a control group.

## 2 Methods

### 2.1 Study population

A total of 147 patients with a diagnosis of AF confirmed by electrocardiography, with chronic oral anticoagulation (CHA_2_DS_2_-VASc ≥2), using warfarin (n = 47) or rivaroxaban (n = 38), were included in the study, as well as healthy individuals (controls, n = 62). At the time of patient screening, the prevailing consensus in the field supported the use of the CHA_2_DS_2_-VASc score, which dictated the choice of these parameters. More recently, however, the CHA_2_DS_2_-VA score has emerged as superior. Patients with AF were recruited from the outpatient clinics of Hospitals Lifecenter, Semper and Ipsemg (Belo Horizonte, Minas Gerais, Brazil). Control subjects were recruited from the local community and had no prior diagnosis of AF or use of any anticoagulant therapy.

The participants were excluded if they used any antiplatelet agent, non-steroidal anti-inflammatory drugs, heparin, hormone replacement therapy, antifibrinolytics, amiodarone, verapamil, quinidine, azole antifungals, and ritonavir in the 4 weeks prior to the study. Moreover, subjects with the following clinical conditions were also excluded: current diagnosis of alcohol use disorder; chronic kidney disease (creatinine clearance <30 mL/min); severe dyslipidemia; acquired or hereditary bleeding disorders; liver disease; thyroid disease; infectious, inflammatory, autoimmune, and malignant diseases; pregnancy; puerperium; and breast-feeding.

The present study was approved by the Research Ethics Committees of *Universidade Federal de Minas Gerais* (UFMG–CAAE: 12603413.0.0000.5149), Lifecenter, Semper and Ipsemg Hospitals and was performed in accordance to the principles provided in the Declaration of Helsinki. All participants received clear information about the research, read and signed the written Informed Consent, before any study procedures.

### 2.2 Biological samples

Venous blood samples were collected in tubes containing ethylenediaminetetraacetic acid (EDTA), and 3.2% sodium citrate, and tubes without anticoagulant, after a 12-h fast. Plasma and serum samples were processed by centrifuge at 25 °C for 15 min at 1,100 g, within 4 h of collection. The samples were identified and stored at −80 °C until analysis.

### 2.3 Laboratory characterization

Serum determinations of biochemical parameters total cholesterol (TC), High Density Lipoprotein-cholesterol (HDL-c), Low Density Lipoprotein-cholesterol (LDL-c) and triglycerides were performed using the automatic analyzer Vitros 250, Johnson & Johnson®.

### 2.4 Analysis of oxidative stress parameters

The oxidative status was evaluated by determining, in serum samples, the lipid peroxidation marker malondialdehyde (MDA), which belongs to the class of thiobarbituric acid reactive species (TBARS), according to the protocol by Vasconcelos et al. and adapted by Duarte (Vasconcelos et al., 2007; Duarte, 2012). To determine the antioxidant capacity of serum, the quantification of MTT [3-(4,5-Dimethylthiazol-2γl)-2,5-Diphenyl Tetrazoline Bromide] was performed according to the protocol by Medina et al. adapted by Duarte (Duarte, 2012; Medina et al., 2007). Both TBARS and MTT were measured by spectrophotometry.

### 2.5 Analysis of anti-fibrinolytic parameters

The evaluation of the fibrinolytic profile was performed on citrate plasma samples by the ELISA method, using the following kits: IMUBIND® Plasma PAI-1 (BIOMEDICA DIAGNOSTICS, USA) and VisuLizeTM TAFI Antigen Kit (AFFINITY BIOLOGICALS INCORPORATED, Canada) for plasma determination of anti-fibrinolytic markers PAI-1 and TAFI, respectively. The samples were analyzed in duplicate, with an intra-assay variation <5%. An internal quality control was used in all assays.

### 2.6 Covariates

The parameters: dyslipidemia, hypertension (HAS), physical activity, age, total cholesterol (TC) were considered as covariates of possible interference in the study results. For the case group, information about such parameters was taken from the patient records, and for the control group, through individual reports. The diagnosis of dyslipidemia is based on the V Brazilian Guideline on Dyslipidemia and Prevention of Atherosclerosis, which consists of: TC ≥ 240 mg/dL; LDL-c ≥ 160 mg/dL; HDL-c < 40 mg/dL; TG (Triglycerides) >200 mg/dL (Xavier et al., 2014). Hypertension followed the criteria of the Brazilian Guidelines on Arterial Hypertension, which consists of: systolic blood pressure >140 mmHg and diastolic blood pressure >90 mmHg (Barroso et al., 2021). Physical activity was considered as: 150–300 min of moderate-intensity aerobic physical activity; or at least 75–150 min of vigorous-intensity aerobic physical activity; or an equivalent combination of moderate and vigorous intensity activity throughout the week for substantial health benefits; according to guidelines on physical activity and sedentary behavior of the World Health Organization (WHO) (WHO, 2020).

### 2.7 Statistical analyses

The sample size was estimated from the mean values of PAI-1 and TAFI levels obtained in the previous studies (Liles et al., 2016; Dungan et al., 2024), respectively, including patients with AF and controls. MTT and TBARS were not used due to the absence of previous studies including them in the comparison between the two groups. The sample size calculation was performed using T-test between two independent groups. The values considered were: power = 0.80; confidence interval = 0.95. The software used was OpenEpi. The ratio 1:1 case/control resulted in at minimum 34 individuals in each group.

All data were subjected to descriptive analysis and continuous variables were analyzed for normal distribution using the Shapiro-Wilk test. Student’s t and Mann-Whitney tests were used to compare two groups (AF vs. controls) for normally or not normally distributed variable, respectively. To compare three groups (warfarin vs. rivaroxaban vs. controls), analysis of variance - ANOVA and post-hoc LSD were used for normal variables. Kruskal–Wallis test, followed by Bonferroni correction were used for non-normal variables. In order to discount the interference effect of confounding variables in the comparison of biomarkers between groups, a multivariate linear regression analysis was performed in two stages. First, a model including all confounding variables was made in order to assess the relationship with biomarkers. In a second step, the variables that presented p < 0.20 were included in the final linear regression model along with groups and biomarkers. Statistical analyzes were performed using the Statistical Package of the Social Sciences (SPSS) version 17.0. Values of p < 0.05 were considered as significant effects. The GraphPad Prism® software version 8.0 (GraphPad Software, La Jolla, CA, USA) was used to prepare the graphs.

## 3 Results

### 3.1 Demographic, clinical and laboratory characteristics

Table 1 presents demographic, clinical and laboratory characteristics of participants. There were no differences between groups regarding smoking, type 2 diabetes mellitus, use of metformin and alcohol consumption. The patients using rivaroxaban are older than the patients who use warfarin and the individuals in the control group. Hypertension was more frequent in warfarin and rivaroxaban treatments regarding to the control group. Dyslipidemia and statin use were more frequent in patients using warfarin. There was no significant difference between patients on warfarin or rivaroxaban group regarding the CHA_2_DS_2_-VASc score. Clearly, physical activity was more frequent in the control group when compared to those treated with warfarin or rivaroxaban. TC levels differed only between the control and rivaroxaban groups with higher levels in control group.

**TABLE 1:** Demographic, clinical, and laboratory characterization of participants of the study.

Parameter	Control (n = 62)	Warfarin (n = 47)	Rivaroxaban (n = 38)	*p*	*p* ^*1*^	*p* ^*2*^	*p* ^*3*^
‡‡Age in years	69 (9)	70 (13)	77 (15)	0.002*	0.565	0.001*	0.010*
Sex							
Female, n (%)	38 (61.3%)	21 (44.7%)	18 (47.4%)	0.176	​	​	​
Male, n (%)	24 (38.7%)	26 (55.3%)	20 (52.6%)	​	​	​	​
Smoker, n (%)	2 (3.2%)	1 (2.1%)	3 (7.9%)	0.371	​	​	​
HAS, n (%)	31 (27.9%^)^	43 (38.7%)^a^	37 (33.3%)^a^	<0.001*	​	​	​
DM2, n (%)	10 (16.1%)	15 (31.9%)	10 (26.3%)	0.146	​	​	​
Dyslipidemia	23 (39%)^b^	32 (68.1%)^a^	16 (42.1%)	0.007*	​	​	​
CHA2DS2-VASc	-	3 (2.5–4)	4 (3–4)	0.776	​	​	​
Physical activity	60 (98.4%)^a^	8 (17.8%)^b^	11 (28.9%)^b^	<0.001*	​	​	​
Alcohol consumption	17 (27.4%)	8 (17%)	13 (34.2%)	0.185	​	​	​
†Total cholesterol (mg/dL)	179.3 ± 36.8	178.5 ± 37.2	178.4 ± 28.6	0.032*	0.235	0.009*	0.151
†LDL-c (mg/dL)	96.6 ± 26.7	89.2 ± 31.9	93.1 ± 20.2	0.190	-	-	-
†HDL-c (mg/dL)	53.1 ± 16.2	56.0 ± 15.9	60.6 ± 14.7	0.242	-	-	-
†Triglycerides (mg/dL)	147.2 ± 49.7	166.5 ± 84.5	123.4 ± 52.4	0.357	-	-	-

Results are presented as †mean and standard deviation (parametric variables), ‡‡median and interquartile range (non-parametric variables) or frequency (absolute and percentage). Chi-square test with residual analysis: a more frequent, b less frequent. Post-hoc LSD or Bonferroni test for multiple comparisons of all pairs of means: p1: Control versus Warfarin; p2: Control versus Rivaroxaban; p3: Warfarin versus Rivaroxaban. Significant Values *: p < 0.05. Abbreviations: SAH, Systemic arterial hypertension; DM2, Type 2 Diabetes Mellitus; LDL, Low Density Lipoprotein; HDL, High Density Lipoprotein; ALT, Alanine Aminotransferase; AST, Aspartate Aminotransferase; GGT, Gamma Glutamyl Transferase; PCR, C-reactive protein.

### 3.2 Analysis of oxidative stress markers

**FIGURE 1:**
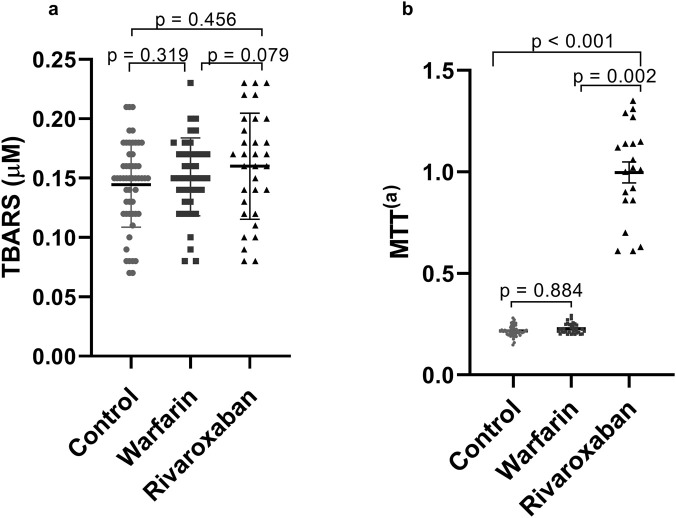
Levels of oxidative stress markers TBARS **(a)** and MTT **(b)** between control, warfarin and rivaroxaban groups. **(a)** TBARS levels compared between control (n = 62), warfarin (n = 47) and rivaroxaban (n = 38) groups; **(b)** MTT absorbance values compared between control (n = 62), warfarin (n = 47) and rivaroxaban (n = 38) groups. P-value <0.05 as significant. Abbreviations: TBARS = thiobarbituric acid reactive species. MTT = 3-4,5-dimethyl-thiazol-2-yl-2,5-diphenyltetrazolium bromide.

The comparative analysis of oxidative stress markers across the control, warfarin, and rivaroxaban groups is presented in Figure 1. No statistically significant differences in Thiobarbituric Acid Reactive Substances (TBARS) concentrations were observed among the groups (p > 0.05, Figure 1a). This lack of significant association persisted after adjustment for potential confounding variables in a multiple linear regression model (data not shown).

In contrast, a significant difference in MTT absorbance was detected. The rivaroxaban group demonstrated markedly higher absorbance values [1.290 (1.040)] compared to both the control group [0.217 (0.029)] (p < 0.001) and the warfarin group [0.212 (0.066)] (p = 0.002, Figure 1b). The statistical significance of these comparisons was maintained following adjustment for confounding variables (Supplementary Table S1).

### 3.3 Analysis of antifibrinolytic parameters

**FIGURE 2:**
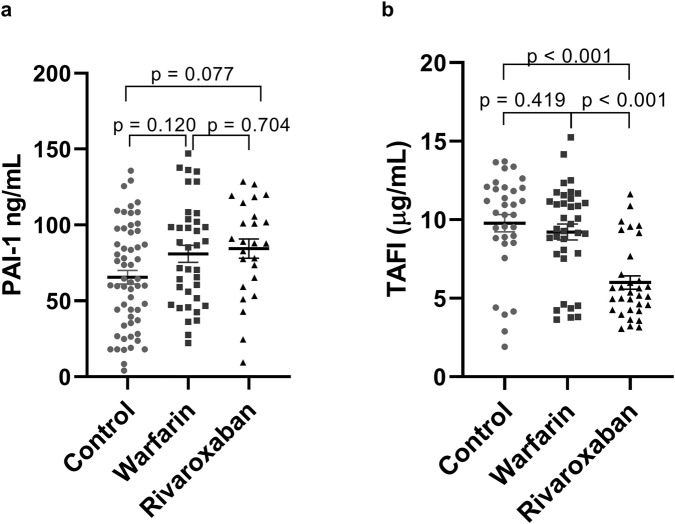
Levels of fibrinolytic markers PAI-1 **(a)** and TAFI **(b)** between control, warfarin and rivaroxaban groups. **(a)** PAI-1 levels compared between control (n = 62), warfarin (=47) and rivaroxaban (n = 38) groups; **(b)** TAFI levels compared between control (n = 62), warfarin (n = 47) and rivaroxaban (n = 38) groups. P-value <0.05 as significant. Abbreviations: PAI-1, plasminogen activator inhibitor-1; TAFI, thrombin activatable fibrinolysis inhibitor.

Plasma levels of the antifibrinolytic biomarker PAI-1 did not differ significantly between the study groups (p > 0.05, Figure 2a). This finding was consistent in the multiple linear regression model accounting for confounders (data not shown).

Analysis of TAFI revealed significant intergroup differences. Serum TAFI levels were significantly lower in the rivaroxaban group (7.4 ± 2.5 μg/mL) compared to both the control group (10.3 ± 2.5 μg/mL; p < 0.001) and the warfarin group (10.0 ± 2.6 μg/mL; p < 0.001, Figure 2b). These differences remained statistically significant after controlling for confounding variables (Supplementary Table S2).

## 4 Discussion

To the best of our knowledge, this study is the first to evaluate oxidative stress parameters and fibrinolytic biomarkers in Brazilian patients with AF undergoing anticoagulant therapy with either warfarin or rivaroxaban. The findings demonstrated that patients treated with rivaroxaban exhibited higher MTT absorbance values, indicating enhanced antioxidant capacity. Additionally, lower TAFI levels were observed in the rivaroxaban group, suggesting a potential inhibitory effect on the expression of anti-fibrinolytic factors.

Emerging evidence suggests that FXa inhibition by rivaroxaban may attenuate the generation of reactive oxygen species (ROS) through indirect mechanisms (Woźniak et al., 2020; Ishibashi et al., 2014; Moñux et al., 2017). Supporting this, Woźniak et al. demonstrated that rivaroxaban and dabigatran confer protective effects on vascular endothelial cells by reducing ROS production and mitigating DNA damage (Woźniak et al., 2020). Although the present study found no significant reduction in oxidative stress as measured by TBARS levels, a distinct antioxidant effect of rivaroxaban was observed, evidenced by increased cell viability in the MTT assay. This finding aligns with research by Ishibashi et al. and Moñux et al., who reported that FXa inhibition with rivaroxaban downregulates oxidative stress-related proteins and promotes an antioxidant milieu in models of abdominal aortic aneurysm (Ishibashi et al., 2014; Moñux et al., 2017). A comparable antioxidant effect has also been documented for edoxaban, another direct FXa inhibitor (Narita et al., 2019). While clinical studies quantifying TBARS in atrial fibrillation are limited, the findings of Abedalqader et al., who observed a reduction in TBARS in a rat model of cardiac abnormalities treated with rivaroxaban, suggest that the impact on this specific biomarker may be model- or context-dependent (Abe et al., 2023).

FXa plays a central role not only in coagulation but also in cellular signaling, primarily through the activation of protease-activated receptors (PARs) (Schiffer et al., 2023). This signaling promotes pro-inflammatory and pro-fibrotic responses, as well as platelet activation (Petzold et al., 2020). Furthermore, FXa interaction with the platelet receptor glycoprotein VI (GPVI) has been linked to a significant increase in oxidative stress (Cammisotto et al., 2019). In support of this mechanism, Pignatelli et al. (2016) observed a marked reduction in soluble GPVI levels in AF patients following 3 months of rivaroxaban therapy, an effect not seen with warfarin (Pignatelli et al., 2016). Collectively, these findings and our data support the hypothesis that the antioxidant effect of rivaroxaban in AF is mediated, at least in part, by the inhibition of FXa-driven cellular pathways. This reinforces the concept that FXa inhibitors exhibit pleiotropic actions, potentially conferring benefits beyond anticoagulation by simultaneously targeting inflammation, fibrosis, and oxidative stress (Narita et al., 2019; Atzemian et al., 2023).

Analysis of fibrinolytic biomarkers revealed significant reductions in serum TAFI levels within the rivaroxaban cohort. The fibrinolytic system is increasingly recognized as a significant predictor of cardiovascular risk (Pang et al., 2017; Sumaya et al., 2020), wherein elevated levels of PAI-1 and TAFI are associated with a prothrombotic state and an increased incidence of thromboembolic events—the primary complication of AF (Kernan et al., 2014).

Our finding of reduced TAFI is consistent with the work of Ma et al., who also reported a significant downregulation of TAFI and other antifibrinolytic markers with rivaroxaban treatment (Ma et al., 2017). A divergence exists, however, as their study, conducted in a rat model, also demonstrated a reduction in PAI-1, which was not observed in our cohort. The absence of a significant change in PAI-1 in our study aligns with the findings of Liles et al., who reported no difference in plasma PAI-1 levels between patients with AF following ablation and a control group (Liles et al., 2016). Furthermore, the broader mechanistic link is supported by Sanda et al., who showed that dabigatran, an anticoagulant that indirectly inhibits FXa, significantly lowered both PAI-1 and TAFI levels and enhanced thrombolytic activity in a murine model (Sanda et al., 2020).

Collectively, these findings substantiate the premise that FXa inhibition can modulate the expression of key antifibrinolytic markers. Nevertheless, the heterogeneity observed across studies underscores the need for further investigation to clarify the specific mechanisms and contextual factors governing these effects.

Moreover, it is well established that FXa contributes to inflammation beyond its coagulation function (Ellinghaus et al., 2016; Chan et al., 2012; Esmon, 2014). Consequently, FXa inhibition by rivaroxaban may induce anti-inflammatory effects in addition to its anticoagulant action (Wu et al., 2015; Kondo et al., 2018; Martins et al., 2020). Recent studies by Miyazawa et al. and Kirchhof et al. further demonstrated that rivaroxaban favorably modulates fibrinolytic and inflammatory markers in patients with AF (Miyazawa et al., 2018; Kirchhof et al., 2020).

Interestingly, some studies suggest an inverse relationship between TAFI levels and inflammation (Pang et al., 2017; Komnenov et al., 2015). For instance, Pang et al. found a strong negative correlation between TAFI and pro-inflammatory cytokines [Interleukin-1β, Interleukin-6, Tumor Necrosis Factor (TNF-α)], procalcitonin (PCT), and C-reactive protein (CRP) in patients with acute coronary syndrome (Pang et al., 2017). These findings highlight the need for further studies to better elucidate the interplay between fibrinolytic and inflammatory pathways in AF patients treated with rivaroxaban, as such investigations remain limited to date.

Several limitations of this study warrant consideration. First, the control cohort was recruited from public gyms, which may have introduced a selection bias, as these individuals likely possess a higher baseline level of physical activity compared to the patients in the anticoagulant groups. Second, the methodological approach for assessing TAFI quantified only the total antigen concentration and did not differentiate between the active (TAFIa) and inactive (TAFIi) isoforms. Given the thermal instability and functional heterogeneity of TAFI, quantification of the activated form (TAFIa/ai) would offer a more precise representation of its antifibrinolytic activity in various pathophysiological contexts (Tregouet et al., 2009). This methodological constraint, which varies across studies, complicates direct comparisons and may underlie the inconsistent findings reported in the literature. Third, the non-randomized, observational nature of the study design precludes the establishment of causal relationships. Finally, the relatively modest sample size within each anticoagulant group may limit the statistical power and external validity of our conclusions. Consequently, further large-scale, prospective studies are warranted to validate and elaborate upon these preliminary findings.

## 5 Conclusion

The treatment with rivaroxaban increased MTT and reduced levels of TAFI, suggesting that, in addition to its anticoagulant action, the drug has a potential antioxidant and pro-fibrinolytic in the AF population. It emphasizes that further studies are needed for a better understanding of the drug profile in arrhythmia complications.

## Data Availability

The raw data supporting the conclusions of this article will be made available by the authors, without undue reservation.
