# Supplementary material for: Rivaroxaban versus warfarin: differential effects on oxidative stress and fibrinolytic markers in atrial fibrillation
Source: Front Pharmacol. 2026 Jan 19;17:1685101. doi: 10.3389/fphar.2026.1685101 (PMC12862254; doi:10.3389/fphar.2026.1685101)
Supplement: Supplementary file 1 [file Supplementaryfile1.pdf]

TABLE S1. Multiple linear regression model for MTT biomarker and study variables.

Term	Coef	EP Coef	T	P
Constant	1.0318	0.0618	16.70	0.000
Groups				
Rivaroxaban	-	-	-	-
Warfarin	-0.8308	0.0540	-15.40	0.000
Control	-0.8126	0.0674	-12.06	0.000
Hypertension				
Not	0.0118	0.0541	0.22	0.828
Dyslipidemia				
Not	-0.0156	0.0423	-0.37	0.713
Physical Activity				
Not	0.0029	0.0723	0.04	0.968

TABLE S2. Multiple linear regression model for TAFI biomarker and study variables.

Term	Coef	EP Coef	T	P
Constant	1.14	3.83	0.30	0.767
Age	-0.0100	0.0405	-0,25	0.806
Total cholesterol	0.03162	0.00931	3.40	0.001
Rivaroxaban	-	-	-	-
Warfarin	3.141	0.750	4.19	0.000
Control	3.846	0.847	4.54	0.000
Hypertension				
Not	-0.508	0.833	-0.61	0.544
Dyslipidemia				
Not	0.663	0.605	1.10	0.276
Physical Activity				
Not	0.303	0.846	0.36	0.721
